# Glandular Odontogenic Cyst in Maxilla: A Case Series

**Published:** 2018-06-30

**Authors:** Bidhata Ojha, Dipshikha Bajracharya, Subrata Bhattacharyya, Radha Baral, Saurabh Roy, Sumit Singh, Bikash Desar

**Affiliations:** 1 Department of Oral and Maxillofacial Pathology, Kantipur Dental College and Teaching Hospital, Kathmandu, Nepal; 2 Department of Oral and Maxillofacial Surgery, Kantipur Dental College and Teaching Hospital, Kathmandu, Nepal

**Keywords:** *histology*, *immunohistochemistry*, *odontogenic cyst*

## Abstract

Glandular odontogenic cyst is rare phenomenon with 0.012% to 0.03% frequency of all jaw cysts and worldwide prevalence of 0.17%. Diagnosis of Glandular odontogenic cyst, well known for its aggressive growth potential and high rate of recurrence, is very crucial. This report presents cases of two 50-year old individuals with Glandular odontogenic cyst presenting as a radiolucent lesion of maxilla. Final diagnosis was made on the basis of histopathological features and further confirmed by immunohistochemical analysis.

## INTRODUCTION

Glandular odontogenic cyst was first documented as ‘Sialo-odontogenic cyst’ by Padayachee, Van Wyk and by Gardner et al. as ‘Glandular odontogenic cyst’(GOC).^1^ Having low frequency of 0.012–0.03%, GOC mostly occurs in fifth decade of life with anterior mandible being common site which has slight preponderance for male.^2^ Lesions are usually asymptomatic and show unilocular or multilocular radiopacities.^3^ Herein, we present two case reports of GOC of maxilla.

## CASE REPORT 1

A 50 years old male patient complained of swelling on palatal region and left side of nose for four months. The swelling was asymptomatic, fluctuant and associated with purulent discharge from nose.

**Figure 1.**
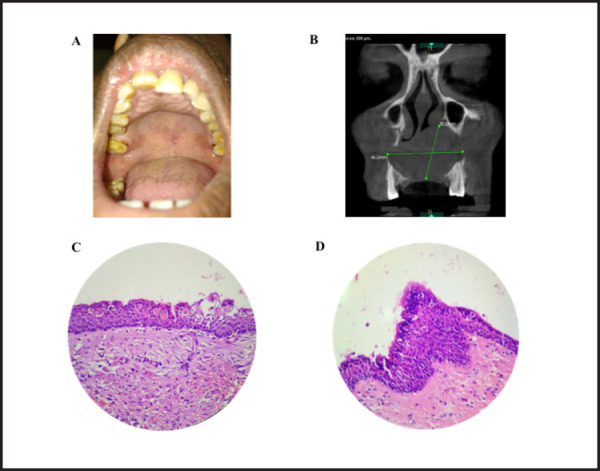
A showing intraoral swelling, B showing unilocular radiolucency involving maxilla. C showing cystic space lined by non-keratinized stratified squamous epithelium with some areas showing ciliated epithelium of variable thickness and numerous mucous cells. D showing plaque-like thickening with mucous cell. (H and E section 400X)

Examination revealed facial asymmetry on the left side with infra orbital swelling extending above the naso-labial fold to the malar prominence laterally. Deviation of nose towards right was observed. Intraoral swelling on the labial gingiva in relation to 21, 22, 23 with erythematous overlying mucosa was noticed (Figure. 1A). Swelling was fluctuant with no discharge and was non-tender on palpation. Vitality test revealed non-vital 21, 22 and 23.

CBCT revealed unilocular radiolucency measuring 30.6 × 40mm extending from roof of palate to floor of nasal cavity. Radiolucency involved nasal septum with its deviation (Figure. 1B). Incisional biopsy revealed cystic space lined by non-keratinized stratified squamous epithelium of variable thickness. Some areas of epithelium showed mucous cells. Based on these features, histopathological differential diagnosis of nasopalatine cyst and glandular odontogenic cyst were given.

The lesion was enucleated and the histopathological examination revealed cystic space lined by non-keratinized stratified squamous epithelium of variable thickness with some areas showing ciliated epithelium. Epithelium contained numerous mucous cells with areas showing plaque-like thickening. Connective tissue showed loosely arranged bundles of collagen fibers with extravasated RBCs (Figure. 1C and 1D).

Immunohistochemistry, Ki-67 labelling index was 70% positive in basal and supra basal layers, <1% in upper layers of epithelial lining. Strong positivity for CK 19 in columnar to cuboidal epithelial lining cells further supported its odontogenic origin (Figure 3A and 3B).

Based on the histopathological features and immunohistochemical findings, final diagnosis of GOC was given.

## CASE REPORT 2

A 50 years old female patient complained of swelling and pain in upper left posterior region for 3 months. Swelling extended from distal of 25 to distal of 27. Swelling was firm and non-tender.

**Figure 2.**
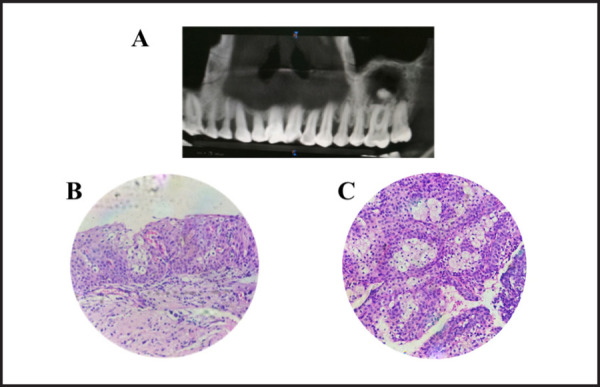
A showing unilocular radiolucency extending from distal of 25 to distal of 27 with diffuse radiopacity. B showing cystic space lined by epithelium of variable thickness. Some areas of epithelium show 2 to 3 layers consisting goblet cells, and some areas show mucous cells. C showing connective tissue area with aggregates of mucous cells with mucous secretion. (H and E section 400X)

CBCT revealed circumscribed round radiolucency of about 2 × 2.5cm with diffuse radio opacity in the center extending from mesial root of 26 to distal root of 27 (Figure 2A).

**Figure 3.**
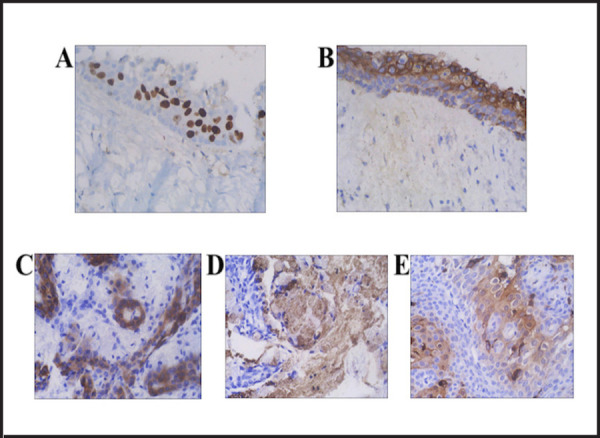
A showing Ki-67 labelling index, 70% in basal and supra basal layers. <1% in upper layers of epithelial lining. B showing strong positivity of CK 19 in columnar to cuboidal epithelial cells and to a lesser extent in non-keratinized squamous epithelial cells. C showing CK5/6 positive in many epithelial cells. D and E showing focal positivity of MUC5AC and S100 in few cells.

Histopathological examination of enucleated sample revealed cystic space in tissue section lined by epithelium of variable thickness. Some areas of epithelium were 2 to 3 layers of goblet cells along with some areas showing mucous cells. The connective tissue stroma was composed of loosely arranged collagen fibers with plump fibroblast. Deeper connective tissue area showed aggregates of mucous cells with mucous secretion. Based on the histological features, differential diagnosis of mucoepidermoid carcinoma (MEC) and GOC were given (Figure. 2B and 2C).

Immunohistochemistry revealed CK 5/6, CK 7, p63 and Epithelial Membrane Antigen (EMA) positive in many epithelial cells. Only few cells showed positivity for S-100, MUC5AC. The cells were negative for Smooth Muscle Actin (SMA) and Calponin. Ki-67 labelling index was 10% (Figure. 3C,3D and 3E). As cells were positive for CK 7 and negative for MUC5AC, a marker of MEC, final diagnosis of GOC was given.

## DISCUSSION

GOC is a developmental cyst with epithelial features simulating salivary gland or glandular differentiation (WHO, 2017).^4^ It is a rare lesion occurring exclusively in jaws, with mandible involved in about 75% of cases whereas lesions tend to occur anteriorly in maxilla which was similar in our case. Lesions are very aggressive with 21–30% recurrence rate.^3,5^ In radiographs, GOC reveal well-defined unilocular or multilocular radiolucent scalloped-bordered lesions, which are associated with roots of multiple teeth causing their displacement or root resorption^4^ which was comparable with our case.

WHO (2017) enlists 10 histopathological criteria for diagnosing GOC: lining epithelium of variable thickness from flattened squamous or cuboidal cells to thick stratified squamous epithelium, focal presentation of cuboidal to low columnar cells which are suggestive of hob nail cells. Intraepithelial microcysts formation, luminal cells with apocrine metaplasia, basal and parabasal layer of clear cells, epithelial papillary projection into the lumen with presence of mucous cells, spheres. Presence of ciliated cells, and multiple cystic compartment.^4^

On Immunohistochemistry, GOC shows strong positivity for bcl-2 in basal, supra basal cell layers and CK 7, 8, 19 suggesting its odontogenic origin.^6^ Differential diagnosis includes lateral periodontal cyst and central mucoepidermoid carcinoma (CMEC). The former lesion lacks ciliated epithelium with duct like spaces and mucous cells. The latter expresses strong positivity for CK18 and Maspin, and lacks superficial cuboidal cells, epithelial whorls, ciliated cells and intraepithelial microcysts.^3^ Enucleation is the treatment of choice with regular follow-up for 3 to 5 years.^2^ To conclude, diagnosis of GOC must include careful evaluation of each details as the features are similar to MEC. Involvement of immunohistochemistry should be considered for confirmatory diagnosis. Regular follow up of patient is mandatory as the lesion has high recurrence rate.

## References

[1] Chandolia B, Bajpai M AM. (2017). Glandular Odontogenic Cyst. J Coll Physicians Surg Pak..

[2] Li L, Singh P, Ping J, Li X, Li L, Singh P (2016). Glandular odontogenic cyst of posterior maxilla : A rare
entity. Int J case Rep images(IJCRI).

[3] Frazier JJ, Flint DJ. (2017). Glandular odontogenic cyst of the anterior maxilla in a 13-year old male: a
rare case of location and age. J Oral Med Surg..

[4] El-Naggar AK, Chan JKC, Grandis JR, Takata T, Slootweg PJ. (2017). WHO classification of head and neck tumours.

[5] Maleki L, Hekmatimoghadam S, Tabatabaei S, Firouzabadi AH, Azam AN. (2016). Glandular odontogenic cyst of the mandible. Journal of Case Reports in Practice (JCRP)..

[6] Pires FR, Chen SY, da Cruz Perez DE, de Almeida OP KL. (2004). Cytokeratin expression in central mucoepidermoid carcinoma and glandular
odontogenic cyst. Oral Oncol..

